# Dental Status and Associated Factors in a Dentate Adult Population in Bulgaria: A Cross-Sectional Survey

**DOI:** 10.1155/2012/578401

**Published:** 2012-05-13

**Authors:** Nikola D. Damyanov, Dick J. Witter, Ewald M. Bronkhorst, Nico H. J. Creugers

**Affiliations:** ^1^Department of Prosthetic Dental Medicine, Faculty of Dental Medicine, Medical University-Sofia, 1, Georgi Sofiiski Boulevard, 1431 Sofia, Bulgaria; ^2^Department of Oral Function and Prosthetic Dentistry, College of Dental Science, Radboud University Nijmegen Medical Centre, 25 Philips van Leydenlaan, 6525 EX Nijmegen, The Netherlands; ^3^Department of Preventive and Restorative Dentistry, College of Dental Science, Radboud University Nijmegen Medical Centre, 25 Philips van Leydenlaan, 6525 EX Nijmegen, The Netherlands

## Abstract

This study aimed to determine variations in the dental status of a dentate adult population in terms of “decayed,” “missing,” and “filled” teeth in relation to several sociodemographic and behavioral factors. Quota sampling was used to draw 2531 subjects aged 20 years and over. Data were collected by means of a questionnaire and an oral examination. Multiple logistic regression analyses were performed to observe associations between “decayed,” “missing,” and “filled” teeth and the factors of interest. The mean numbers of “decayed,” “missing,” and “filled” teeth were 2.2, 6.7, and 4.9, respectively. Molar teeth were significantly more often “missing” than premolar and anterior teeth. Age, gender, education, and tooth brushing revealed most noticeable associations. Increasing age was associated with a lower chance of having “decayed” and “filled” teeth, but with a higher chance of having “missing” teeth. Females were more likely to have “missing” and “filled” teeth. Higher education was associated with a lower chance of having “missing” teeth. More frequent tooth brushing was associated with a lower chance of having “decayed” and “missing” teeth, but with a higher chance of having “filled” teeth. These risk indicators should be considered in prevention program planning if reduction of tooth loss is to be achieved.

## 1. Introduction

The decline of dental caries and tooth loss has been well documented in Europe [1]. Nevertheless, considerable variations in dental health status still exist between and within countries. In some Central and Eastern European countries the prevalence of dental caries and edentulism is still high [2, 3]. Furthermore, even in affluent Western European societies with sophisticated dental health care delivery systems, the most deprived population groups experience the worst dental health [4, 5]. Dental literature has shown variations in dental health to be determined by a wide range of personal, social, economic, and environmental factors. Correlates of dental health have been studied in various settings across Europe, mostly in children and adolescents, and less so in adults. Available studies of adults have shown that population groups with lower education, poor income, or low occupational status tend to have more dental caries [6, 7] and less remaining teeth [8]. Furthermore, variations in dental health status have been associated with unfavorable oral health behavior [9, 10] and unhealthy lifestyle [11], as well as with demographic factors such as age, gender, or place of residence [12, 13].

Little is known about adult oral health in Bulgaria, especially with respect to factors associated with dental health status. The last national oral health survey, conducted in 1989, showed that the prevalence of dental caries and tooth loss among adults is high [14]. The prevalence of caries disease did not differ significantly between the genders and between urban and rural residents; other potentially associated factors were not investigated. At that time, the health care system in Bulgaria was integrated into a centralized state organization and oral health care services were provided free of charge for all citizens [15]. Since 1989, however, radical political and economic changes have occurred in the country, including privatization of oral health care services [16]. These new circumstances may have created oral health differences among the adult population, but this issue has never been investigated in Bulgaria.

In a recently proposed risk factor model for dental caries, distal socioenvironmental factors shape more proximal behavioral factors that are associated with an adverse outcome [17]. An outcome variable frequently used in prediction studies is the mean number of decayed (D), missing (M), and filled (F) teeth (DMFT index). This approach, however, overlooks the importance of the index components, each of them representing different dimension of the disease process. Another problem is that most of the available studies do not account for the presence of site–specific risks, for example, difference in risks even within the same oral cavity [18]. 

The aim of this study was to identify sociodemographic and behavioral factors associated with decayed, missing, and filled teeth in a dentate adult population in Bulgaria, accounting for differences between anterior, premolar, and molar dental regions. It was hypothesized that there would be no variations in dental health status between genders, between different residential areas, and between different social groups. The present study reports first findings from a survey which purpose was to collect cross–sectional data regarding correlates of dental and prosthetic status in Bulgarian adults, including assessment of oral function and oral health–related quality of life in subjects with a reduced dentition. 

## 2. Material and Methods

### 2.1. Sample Construction

The target population for this cross–sectional survey was adults aged 20 years and over living in Bulgaria. To calculate the sample size, it was decided that the size of the sample must allow for multiple logistic regression with at least 12 independent variables. This implied that at least 120 observations of the least prevalent part of a dichotomous variable are necessary [19]. Using a 5% prevalence rate as a worst-case scenario, a total sample size of 2400 was required to attain the 120 observations needed. A quota sampling method was adopted for this survey and quota units were established with regard to some demographic (settlement), social (occupational status), and dental (dentition) characteristics. Four groups of settlements were defined based on their population size and administrative functions: the capital city, main urban centers, towns, and rural settlements (a small town or a village). Occupational status was expressed in terms of the nine occupational categories (excluding military forces) provided by the European Union variant of the International Classification of Occupations (ISCO 88 (COM)) [20], with a supplementary category for retired subjects. A dentition was classified as complete, interrupted, or shortened on the basis of morphological characteristics.

It was determined to draw 1/6 of the sample from the capital city and the rest equally distributed between the other three groups of settlements, aiming at broad geographic representation and equal distribution of subjects between occupational categories. With respect to dentition quota, a minimum of 256 shortened dentitions were the only preconceived determination, since the prevalence of this dentition group in the population was expected to be the lowest. Recruitment of participants continued until broad geographic representation had been achieved and the requirements for sample size and shortened dentition quota had been satisfied. 

A total of 16 settlements were selected for this survey: the capital city Sofia (population 1.162.898), main urban centers (population between 347.600 and 140.710), towns (population between 86.978 and 22.267), and rural settlements (population between 9.044 and 1.198) [21]. The main approach to recruit subjects within the selected settlements was to gain access to groups, such as in public or private factories and institutions. Relevant authorities in the survey sites were asked for assistance in the recruitment of subjects. Subjects were recruited from a total of 24 factories and institutions, which represented various service and industrial sectors (e.g., public administration, education, trade, manufacturing, construction, transport). Recruitment of workers was carried out in the framework of the annual statutory occupational health examinations. Retired subjects were recruited from local health care centers in rural settlements and a home for elderly people in the capital city.

Data for this study were collected between October 2006 and January 2010. The requirements for sample size and completion of quotas were sufficiently satisfied when 2644 subjects were examined. Of all eligible subjects available for inclusion in the survey, 313 refused participation. Following exclusion of 113 edentulous subjects, data from 2531 dentate subjects were analyzed for the present study.

### 2.2. Data Collection

The Ethical Committee of the Medical University–Sofia approved this study (number 299/15.05.2007). The research was carried out in compliance with the Helsinki Declaration. Verbal consent was obtained from each subject prior to data collection. Data for this study were collected by means of a self–administered questionnaire and an oral examination. The questionnaire contained items regarding a number of sociodemographic and behavioral variables, including age, gender, level of education, occupational status, household income, and oral health behavior. All oral examinations were done by one calibrated examiner in natural light using a mirror and a dental probe, with the subject seated in an ordinary chair. A headlight was used when the natural light was felt to be insufficient. The examiner was calibrated against experienced researchers at the beginning and halfway through the data collection process by examining convenience samples of 10 subjects. The interexaminer agreement for assessing decayed, missing, and filled teeth was very good in both sessions (all Cohen's kappa statistics ≥ 0.95).

### 2.3. Independent Variables

For this study, education, occupation, and income were selected as distal (sociocultural) independent variables; dental visits and tooth brushing patterns were employed as more proximal (behavioral) independent variables. Educational attainment was determined on the basis of years of education completed and classified as lower (≤9 years), middle (>9 years ≤12), or higher (>12 years) in accordance with the International Standard Classification of Education (ISCED) [22]. Occupational status was recorded as reported by subjects. To facilitate interpretation of data, the nine occupational categories of ISCO 88 (COM) were collapsed into 3 groups: managers, professionals, and technicians were combined to form the “professionals” group; clerks, service, agricultural, and craft workers formed the “intermediate” group; operative and elementary occupations formed the “workers” group. Retired subjects formed a separate fourth group, because we had considered that this would better reflect the social reality in the country rather than allocating retired subjects to occupational categories based on their last occupation. Combined household income was self–rated by the subjects on a 5–point scale and subjects were assigned to 3 income categories: high (income rated as “excellent” or “very good”), medium (income rated as “good”), or low (income rated as “fair” or “poor”).

Dental visits were considered as regular if subjects reported visiting a dentist on a regular basis (at least once a year); dental visits were considered as irregular if subjects reported less frequent dental visits (less than once a year). Frequency of tooth brushing was scored as follows: two or more times a day; once a day; less than once a day.

### 2.4. Dependent Variables

Following the completion of the questionnaire, subjects received an oral examination and the status of each tooth was recorded as “decayed,” “missing,” “filled,” or “sound.” A tooth was considered “decayed” if primary or secondary caries was detected, or if the tooth was fractured. Caries was assessed by visual inspection, added with tactile inspection with a dental probe if required. Only cavitated lesions with softened surfaces were recorded as caries. Root rests were considered as “decayed,” as well. In doubtful cases, no caries was recorded. A tooth was recorded as “missing” if the tooth was clinically absent. “Filled” was recorded for teeth having a dental restoration without the presence of caries. All present teeth free of caries and restorations were considered “sound.”

### 2.5. Data Analyses

Categorical data are presented as counts and percentages. Continuous data are presented as means ± standard deviation (SD). All measures of dental status were calculated for dentitions comprising 32 teeth. For each subject (i.e., within the same oral cavity), the relative scores for “decayed,” “missing,” and “filled” teeth per dental region (anterior, premolar, or molar) were determined by dividing the number of teeth with the respective status (i.e., “decayed,” or “missing,” or “filled”) by the total number of teeth in each dental region (i.e., “decayed,” “missing,” “filled,” and “sound”). Subsequently, the mean relative scores for “decayed,” “missing,” and “filled” teeth of the molar dental region were compared with the relative scores for “decayed,” “missing,” and “filled” teeth of the anterior and premolar dental regions. Differences in the mean relative scores between the dental regions were tested by paired *t*–tests with 95% confidence interval (CI).

Multiple logistic regression analyses were performed to determine associations between the independent variables and “decayed,” “missing,” and “filled” teeth. To allow for statistical modeling, relevant ratios were calculated, that is, “decayed”/“decayed”+“missing”+“filled” (Decayed_ratio_), “missing”/“decayed” + “missing”+“filled” (Missing_ratio_), “filled”/“decayed”+“missing”+“filled” (Filled_ratio_), and dichotomized because of their skewed distributions. Cut–off points focusing on the extreme values of the ratios were selected: no “decayed” teeth present versus one or more “decayed” teeth present (for Decayed_ratio_); no “missing” teeth versus one or more teeth “missing” (for Missing_ratio_); no “filled” teeth present versus one or more “filled” teeth present (for Filled_ratio_). Odds ratios (ORs) were calculated with 95% CI for Decayed_ratio,_ Missing_ratio,_ and Filled_ratio_ for the whole dentition and for the three dental regions separately. The force entry method was used, that is, all independent variables were entered in the regression models in a single step. Subjects with missing data were excluded from the multiple logistic regression analyses.

To assess the performance of the multiple logistic regression models for having “decayed”, “missing”, and “filled” teeth in the whole dentition and in the three dental regions, positive predictive values (PPVs) and negative predictive values (NPVs) were calculated. A value of *P* ≤ 0.05  was considered as statistically significant. The Statistical Package for Social Sciences version 16 for PC (SPSS Inc., Chicago, IL, USA) was used for the analyses.

## 3. Results

### 3.1. “Decayed,” “Missing,” and “Filled” Teeth

Distribution of dentate subjects in the study sample according to sociodemographic and behavioral characteristics is presented in Table 1. Percentages of subjects having “decayed,” “missing,” or “filled” teeth and mean numbers of “decayed,” “missing,” and “filled” teeth are presented in Table 2. Overall, with increasing age, the mean number of “decayed” and “filled” teeth decreased, while the mean number of “missing” teeth increased. Females showed more “missing” and “filled” teeth than males. Subjects living in urban centers presented with less “decayed” and “missing” teeth, but more “filled” teeth than those living in other settlements. Percentages of “decayed,” “missing,” and “filled” teeth per tooth number are presented in Table 3. Generally, in all age groups, molar teeth appeared to be more often “decayed,” “missing,” and “filled” than premolar and anterior teeth, with this trend being less pronounced and sometimes reversed in the upper jaw. Of all molars, fewer third molars were “decayed” and “filled,” but more were “missing” when compared to first and second molars, with these differences becoming smaller with increasing age. 

The molar region in the upper jaw showed statistically significant more “decayed” and “filled” teeth in comparison to the anterior region, but not to the premolar region (Table 4). The molar region in the lower jaw showed significantly more “decayed” and “filled” teeth than the premolar and anterior regions. Molar regions in both jaws showed significantly more “missing” teeth compared to premolar and anterior regions. The differences were more pronounced in the lower jaw than in the upper jaw. To account for the relative high fraction of “missing” third molars, the mean relative scores for “decayed,” “missing,” and “filled” teeth between the dental regions were compared in a subsequent paired *t*–tests excluding the third molars (not shown in the table). When third molars were excluded, the mean differences in relative scores for “decayed” and “filled” teeth between the regions increased, whereas the mean differences in relative scores for “missing” teeth decreased. As a result, the mean differences in relative scores for “missing” teeth between molar and premolar region in the upper jaw became insignificant (*P* = 0.053). With this exception, molar regions in both jaws showed significantly more “decayed,” “missing,” and “filled” teeth than anterior and premolar regions (*P* < 0.001).

### 3.2. Multiple Logistic Regression Analyses

Age was the only factor in this study that showed significant associations with all dependent variables in the dentition as a whole as well as in each dental region (Tables 5(a) and 5(b)). Females were more likely to have “missing” teeth (OR : 1.87) and “filled” teeth (OR : 1.89) than males in the dentition as a whole (Table 5(a)). On dental region level, females had almost two times higher chance of having “missing” molar teeth than males, but a significantly lower chance of having “missing” anterior teeth (Table 5(b)). With respect to place of residence, subjects living in urban centers demonstrated a significantly lower chance of having “decayed” teeth, whereas subjects living in towns showed significantly higher odds of having “filled” teeth. Subjects living in urban settlements were less likely to have “missing” premolar teeth than rural residents (Table 5(b)). Higher education was associated with a lower chance of having “missing” teeth, while lower education was associated with a lower chance of having “filled” teeth. Regular dental visits and more frequent tooth brushing were associated with a lower chance of having “decayed” teeth and with a higher chance of having “filled” teeth; more frequent tooth brushing was associated with lower odds of having “missing” teeth as well (Tables 5(a) and 5(b)). 

Evaluation of the logistic regression models revealed that the models comprising all dental regions (the whole dentition) produced the highest PPV and NPV, resulting in the highest fraction of correct predictions (FC): 68.6% for “decayed” teeth; 91.8% for “missing” teeth; 87.4% for “filled” teeth. With respect to the logistic regression models for each dental region, the molar region had the highest scores for FC: 63.0% for “decayed” teeth; 90.6% for “missing” teeth; 79.7% for “filled” teeth, followed by the premolar region (67.5% for “decayed” teeth; 75.7% for “missing” teeth; 73.9% for “filled” teeth) and the anterior region (65.2% for “decayed” teeth; 71.4% for “missing” teeth; 69.6% for “filled” teeth). Of all dental status measures employed in the logistic regression models, “missing” teeth had the highest fraction of correct predictions, followed by “filled” and “decayed” teeth.

## 4. Discussion

The present findings demonstrate variations in dental health status among adults in the study sample and therefore the hypothesis can be rejected. Differences in the dental health status were associated with age, gender, place of residence, educational background, occupational status, dental attendance patterns, and tooth brushing frequency. Furthermore, different factors were associated with the different outcome variables and these associations were different for the three dental regions. “Missing” teeth were more prevalent and had higher mean scores than “decayed” and “filled” teeth. The total dental disease experience was mainly determined by the molar teeth (both with or without third molars taken into account) as they were generally more often “decayed,” “missing,” and “filled” than premolar and anterior teeth, which finding concurs with previous reports [23–25].

For the present survey, a quota sampling method was adopted. Although this method attempts to ensure that the sample will be representative of a population for specified criteria or strata, it may not be representative for others [26, 27]. For instance, the recruitment strategy resulted in a disproportionate sample comprising fewer older subjects and females compared to their distribution in the general adult population in Bulgaria. Consequently, outcomes with respect to the prevalence of dental conditions cannot be considered representative for the general population. Nevertheless, the inclusion of a wide spectrum of geographic and socioeconomic activity groupings in the study sample, together with the large sample size, were considered adequate to study factors associated with variations in the dental health status. Accordingly, associations with distal and proximal factors may be inferred to the population, since they are not likely to be sensitive to imbalances in the study sample. Therefore, the principal finding from this study that sociodemographic and behavioral factors modified the dental health profile is considered to be relevant for oral health care planners.

In the present study, all demographic factors showed associations with the dental health status. Age and gender are relevant but not modifiable correlates of dental health. With respect to place of residence, however, the outcomes might be attributed to differences in availability of oral health care services between the settlements included in this study. Indeed, the dentist-to-population ratio greatly varied between the settlements ranging from 1 to 780 in the capital city to 1 to more than 2100 in the rural settlements [28]. A striking finding is that all urban settlements (capital, urban centers, and towns) showed a lower chance of having “missing” premolar teeth than the rural settlements. Given that community water fluoridation has never been implemented in Bulgaria and the low level of naturally occurring fluoride in the drinking water [29], a better understanding of the variations between the settlements requires additional information on other factors related to dental health, such as personal perceptions and attitudes to dental health. 

Of the sociocultural factors included in the regression analyses, education emerged as a stronger risk indicator of dental health status than income and occupation. Other studies have shown a significant social gradient in dental status by occupational class [30] and household income [31]. Nonetheless, direct comparison of the present material with previous studies is hindered by differences in the study designs and age groups considered, as well as by the selection of explanatory and outcome variables. The present findings comply with a previous suggestion that education may play a more important role for dental health than income [32]. 

Dental attendance and tooth brushing were included as indicators of oral health behavior. Regular dental visits and more frequent tooth brushing were associated with a lower chance of having “decayed” teeth and a higher chance of having “filled” teeth. Higher frequency of filled teeth among subjects with more attentive tooth brushing habits was reported in a previous study, where it was suggested that practicing proper oral hygiene could indicate a better attitude towards oral health, which may result in more dental visits and more filled teeth [33]. The same reasoning seems to be applicable for the present study. More frequent tooth brushing was associated with a lower chance of having “missing” teeth, while such correlation could not be demonstrated with respect to dental attendance, which is in contrast to other studies [34, 35]. This confirms that regular dental attendants do not necessarily enjoy advantages over irregular attendants with respect to their total dental disease experience [36]. In a recent oral health survey in Europe, the vast majority (80%) of Bulgarians interviewed in the survey stated that the last time they visited a dentist was for a routine or emergency treatment, while only 20% saw a dentist for a checkup, examination, or cleaning [37]. The present outcomes indicate the need for a shift toward preventive oral health behavior rather than treatment oriented approaches as currently applied in Bulgaria. 

In dental epidemiology, DMF teeth or surfaces have been traditionally used to describe dental status and treatment needs in young populations. The presumption that restorations or extractions of teeth are consequences of dental caries only, however, creates problems when applied to adult populations [38]. Tooth decay does not necessarily lead to a restoration, nor have all absent or restored teeth been decayed [39]. Moreover, in a cross–sectional setting, the study participant must recall the reason for having one or more teeth restored or extracted, which introduces the problem of recall bias. In the present study, only “decayed” teeth estimated the true caries disease experience, whereas “missing” and “filled” teeth were considered as adverse outcomes, not necessarily related to dental caries. The associations with respect to “missing” teeth are of particular importance, since tooth loss can have a substantial influence on oral function and social interaction [40]. Age and female gender were associated with a higher chance of having “missing” teeth, while higher education and more frequent tooth brushing reduced the chance of having “missing” teeth. These associations, however, were not always alike and in the same direction for all dental regions. For instance, the association between tooth brushing and “missing” teeth could not be demonstrated for the premolar region, while the association between high education and “missing” teeth was demonstrated only for the premolar region. Furthermore, the higher chance for females of having “missing” teeth was determined by the molar teeth since there were no differences between the genders for “missing” premolar teeth, and conversely females had a significantly lower chance of having “missing” anterior teeth than males. It can be suggested that despite the fact that females had a higher chance of “missing” (molar) teeth than males, they might still have a better chance to retain a functional dentition. However, the present data provided no information with regard to the functionality of the reduced dentitions. Further investigation is needed to assess the impact of tooth loss on oral function and oral health-related quality of life in relation to sociodemographic and behavioral factors.

## 5. Conclusions

Sociodemographic and behavioral factors modified the dental health profile of Bulgarian adults in this study population. “Missing” teeth were more prevalent and had higher mean scores than “decayed” and “filled” teeth. Molars were more often “missing” than premolar and anterior teeth. Age, female gender, having low education, and less frequent tooth brushing are risk indicators for having “missing” teeth. These indicators should be considered in prevention program planning if reduction of tooth loss is to be achieved.

## Figures and Tables

**Table 1:** Distribution of dentate subjects in the study sample according to sociodemographic (*N*, %)  and sociobehavioral (%) characteristics.

		Education (%)*	Income (%)*			Dental visits (%)*	Tooth brushing (%)*		
		>12 years	High	Middle	Low	Irregular	<1 a day	1 a day	≥2 a day
	*N* (%)								
Total sample	2531 (100)	37	9	39	52	55	8	37	55
Age									
20–44	1102 (44)	31	6	33	61	59	11	42	47
45+	1429 (56)	41	11	44	45	53	5	33	62
Gender									
Male	1516 (60)	31	9	40	51	60	11	44	45
Female	1015 (40)	46	7	39	54	49	3	26	71
Settlement									
Capital	397 (16)	39	8	41	51	49	7	31	62
Urban Centre	716 (28)	46	12	46	42	48	3	33	64
Town	704 (28)	23	9	35	56	60	13	41	46
Rural	714 (28)	40	5	37	58	62	8	39	53
Occupational status									
Professionals	832 (33)	81	11	49	40	44	2	33	65
Intermediate	1034 (41)	17	9	38	53	60	8	37	55
Workers	559 (22)	10	6	31	63	67	14	44	42
Retired	78 (3)	22	0	18	82	46	14	28	58
Unknown	28 (1)	61	11	50	39	40	7	25	68

* Subjects with missing data (3 for education; 19 for income; 1 for dental visits; 12 for tooth brushing) were not considered in the percentage.

**Table 2:** Distribution and mean number ± standard deviation (SD) of “decayed,” “missing,” and “filled” teeth according to sociodemographic characteristics of the study sample.

Groups	Number of subjects	Decayed teeth		Missing teeth		Filled teeth	
		% subjects	Mean (SD)	% subjects	Mean (SD)	% subjects	Mean (SD)
Total sample	2531	67	2.2 (2.9)	91	6.7 (6.4)	87	4.9 (4.0)
Age group							
20–29	468	67	2.3 (2.9)	77	2.3 (2.0)	86	4.8 (4.0)
30–39	649	71	2.5 (3.3)	86	3.4 (2.9)	93	6.2 (4.2)
40–49	609	69	2.3 (2.9)	97	6.9 (5.0)	91	5.5 (4.0)
50–59	606	65	1.8 (2.4)	99	10.4 (6.6)	83	3.9 (3.5)
≥60	199	54	1.8 (2.7)	99	15.7 (8.5)	66	2.2 (2.6)
Gender							
Male	1516	68	2.3 (3.0)	90	6.1 (6.0)	84	4.2 (3.7)
Female	1015	66	1.9 (2.6)	93	7.5 (6.8)	90	5.9 (4.3)
Settlement							
Capital	397	68	2.4 (2.8)	88	6.7 (7.4)	88	5.1 (3.9)
Urban center	716	62	1.8 (2.6)	85	4.0 (4.1)	89	5.2 (3.9)
Town	704	69	2.3 (3.0)	94	7.1 (6.0)	87	4.5 (3.7)
Rural	714	71	2.3 (3.0)	96	9.0 (7.1)	84	5.0 (4.5)
Occupational status							
Professionals	832	61	1.6 (2.0)	91	5.6 (5.2)	91	6.1 (4.3)
Intermediate	1034	72	2.5 (3.2)	89	6.0 (5.9)	87	4.7 (3.8)
Workers	559	71	2.7 (3.3)	94	7.8 (6.5)	82	3.8 (3.5)
Retired	78	42	1.1 (1.8)	99	19.3 (8.5)	68	2.5 (3.2)
Unknown	28	64	1.9 (2.1)	93	6.7 (6.7)	82	6.3 (5.3)

**Table 3:** Percentage “decayed,” “missing,” and “filled” central and lateral incisors (I_1_ and I_2_), canines (C), premolars (PM_1_ and PM_2_), and molars (M_1_, M_2_, and M_3_) according to age (left and right side combined).

	20–29 years			30–39 years			40–49 years			50–59 years			≥60 years		
% teeth being	Decayed	Missing	Filled	Decayed	Missing	Filled	Decayed	Missing	Filled	Decayed	Missing	Filled	Decayed	Missing	Filled
Upper jaw															
I_1_	4.7	0.5	15.2	5.8	1.4	15.9	6.0	5.4	20.8	6.4	13.1	17.6	9.0	32.9	8.3
I_2_	5.9	1.4	10.6	4.9	2.2	15.3	5.8	8.4	18.1	6.5	17.7	14.9	9.0	36.7	10.1
C	2.1	0.7	3.2	4.7	1.2	8.8	7.3	5.4	15.9	6.2	11.6	11.8	10.8	28.4	8.5
PM_1_	8.9	3.1	19.1	10.2	6.9	29.0	9.5	20.4	24.5	7.6	37.2	15.7	7.0	53.0	8.0
PM_2_	6.4	4.2	23.4	11.7	10.9	29.7	9.4	28.5	21.3	6.4	44.6	13.0	5.5	63.8	7.5
M_1_	16.3	4.2	40.3	12.8	12.6	41.1	9.9	28.5	31.9	7.6	43.6	19.7	6.8	61.8	8.8
M_2_	12.0	0.3	23.8	11.0	4.2	33.3	9.8	20.5	28.9	8.3	35.3	19.2	6.5	49.0	12.6
M_3_	8.0	41.7	4.4	7.8	46.1	8.1	6. 0	67.7	6.7	3.0	78.7	4.5	2.3	88.7	2.0
Lower jaw															
I_1_	0.3	0.3	1.0	1.6	1.1	1.1	0.7	5.3	1.8	1.6	13.1	1.8	1.5	31.2	2.0
I_2_	0.4	0.2	0.6	1.2	0.8	1.2	1.1	3.3	2.4	2.5	9.4	2.7	2.3	27.9	1.3
C	0.2	0.1	0.2	2.2	0.2	1.4	2.7	1.3	4.2	3.8	4.8	5.0	4.0	16.6	5.5
PM_1_	2.8	0.5	2.6	5.3	2.4	9.0	6.7	8.9	13.1	5.4	16.0	14.4	5.3	35.4	9.0
PM_2_	6.2	3.0	14.4	7.9	6.5	24.9	9.5	18.8	19.4	5.9	31.0	14.6	4.0	47.2	7.3
M_1_	16.8	9.8	44.1	13.0	23.6	40.0	9.4	42.8	25.9	6.7	58.0	14.6	4.5	71.4	7.5
M_2_	16.1	2.6	34.1	15.2	9.2	38.0	11.1	24.5	30.9	8.4	40.5	17.5	6.0	63.3	8.3
M_3_	7.4	40.9	5.2	8.6	41.5	13.3	7.9	56.3	10.9	6.1	66.0	8.1	3.8	78.6	3.5

**Table 4:** Relative scores (%) for “decayed,” “missing,” and “filled” teeth in molar (M), premolar (PM), and anterior (A) dental region and mean difference (%) of relative scores between the dental regions.

Relative scores	%	Comparison	Mean difference (%)	95% CI	*P* value
Upper jaw					
Decayed					
M	8.9				
PM	8.7	M–PM	0.2	−0.5–0.9	0.517
A	5.9	M–A	3.0	2.3– 3.7	**<0.001**
Missing					
M	35.4				
PM	23.3	M–PM	12.1	11.1–13.1	**<0.001**
A	8.1	M–A	27.3	26.3– 28.3	**<0.001**
Filled					
M	20.7				
PM	21.0	M–PM	−0.3	−1.3–0.8	0.627
A	13.9	M–A	6.9	5.8– 8.0	**<0.001**
Lower jaw					
Decayed					
M	10.0				
PM	6.2	M–PM	3.7	3.0–4.5	**<0.001**
A	1.7	M–A	8.3	7.6– 9.0	**<0.001**
Missing					
M	38.3				
PM	13.7	M–PM	24.6	23.6–25.6	**<0.001**
A	5.2	M–A	33.1	32.0– 34.1	**<0.001**
Filled					
M	22.1				
PM	14.0	M–PM	8.1	7.1–9.2	**<0.001**
A	2.1	M–A	20.0	19.0–21.0	**<0.001**

**Table:** (a) Odds ratios (OR) of “decayed,” “missing,” and “filled” ratios* with 95% confidence interval (CI) for the whole dentition.

	Decayed		Missing		Filled	
	OR	95% CI	OR	95% CI	OR	95% CI
Age^†^	**0.981**	**0.972–0.989**	**1.108**	**1.087–1.13**	**0.978**	**0.967–0.989**
Female^a^	1.08	0.88–1.31	**1.87**	**1.31–2.67**	**1.89**	**1.40–2.55**
Capital^b^	1.02	0.75–1.38	0.82	0.47–1.43	1.28	0.84–1.93
Urban centre^b^	**0.66**	**0.51–0.84**	0.69	0.42–1.14	1.18	0.82–1.69
Town^b^	0.83	0.65–1.07	0.89	0.51–1.56	**1.45**	**1.04–2.02**
Education high^c^	0.89	0.70–1.13	**0.66**	**0.45–0.96**	1.09	0.75–1.57
Education low^c^	1.31	0.77–2.21	2.23	0.29–17.39	**0.35**	**0.22–0.57**
Professionals^ d^	**0.74**	**0.57–0.96**	1.17	0.77–1.77	1.31	0.88–1.95
Workers^d^	0.84	0.66–1.07	1.05	0.67–1.66	0.99	0.73–1.35
Retired^d^	**0.36**	**0.19–0.66**	0.17	0.02–1.51	0.75	0.36–1.54
Income high^e^	0.89	0.65–1.23	1.03	0.62–1.70	0.80	0.48–1.31
Income low^e^	1.19	0.98–1.44	0.94	0.67–1.31	0.77	0.58–1.02
Regular visits^f^	**0.47**	**0.39–0.57**	1.01	0.74–1.39	**1.91**	**1.44–2.54**
Tooth brushing^†^	**0.75**	**0.64–0.88**	**0.64**	**0.47–0.86**	**1.33**	**1.09–1.62**

* Dichotomized Decayed_ratio_ (“decayed”/“decayed”+“missing”+“filled”), Missing_ratio_ (“missing”/“decayed”+“missing”+“filled”), Filled_ratio_ (“filled”/“decayed”+“missing”+“filled”). Cut–off points: no “decayed” teeth present versus one or more “decayed” teeth present (Decayed_ratio_); no “missing” teeth versus one or more teeth “missing” (Missing_ratio_); no “filled” teeth present versus one or more “filled” teeth present (Filled_ratio_). ^†^Age and Tooth brushing—numerical variables. References (OR = 1) respectively: ^“a”^Male; ^“b”^Rural settlements (small towns and villages); ^“c”^Education middle; ^“d”^Occupational status intermediate; ^“e”^Income middle; ^“f”^Irregular visits. Bold figures indicate significant relationship.

**Table:** (b) Odds ratios (OR) of “decayed,” “missing,” and “filled” ratios* with 95% confidence interval (CI) for anterior, premolar, and molar dental regions.

	Decayed		Missing		Filled	
	OR	95% CI	OR	95% CI	OR	95% CI
Anterior region						
Age^†^	**0.989**	**0.979–0.999**	**1.071**	**1.058–1.084**	**0.981**	**0.97–0.992**
Female^a^	0.83	0.65–1.06	**0.74**	**0.57–0.96**	**2.42**	**1.86–3.14**
Capital^b^	0.96	0.68–1.37	0.83	0.56–1.23	1.27	0.88–1.85
Urban centre^b^	**0.65**	**0.47–0.90**	0.70	0.49–1.01	1.25	0.89–1.76
Town^b^	0.81	0.60–1.08	0.81	0.60–1.11	**1.64**	**1.20–2.24**
Education high^c^	0.75	0.55–1.03	0.75	0.53–1.06	1.36	0.97–1.89
Education low^c^	1.48	0.89–2.44	1.66	0.94–2.91	0.69	0.41–1.15
Professionals^d^	0.85	0.61–1.19	0.97	0.67–1.40	1.26	0.89–1.79
Workers^d^	1.10	0.82–1.45	1.19	0.88–1.61	0.83	0.62–1.11
Retired^ d^	0.86	0.44–1.69	1.05	0.49–2.23	0.69	0.35–1.36
Income high^e^	0.77	0.50–1.21	0.97	0.59–1.59	1.06	0.67–1.69
Income low^ e^	0.89	0.70–1.13	1.20	0.93–1.55	1.03	0.81–1.33
Regular visits^f^	**0.50**	**0.40–0.64**	0.96	0.75–1.24	**1.95**	**1.52–2.49**
Tooth brushing^†^	**0.77**	**0.64–0.92**	**0.72**	**0.59–0.87**	**1.31**	**1.09–1.58**
Premolar region						
Age^†^	**0.976**	**0.967–0.984**	**1.095**	**1.083–1.106**	**0.958**	**0.949–0.968**
Female^a^	0.87	0.71–1.07	1.03	0.82–1.30	**1.44**	**1.14–1.81**
Capital^b^	1.09	0.80–1.47	**0.59**	**0.42–0.83**	1.34	0.95–1.88
Urban centre^b^	**0.65**	**0.50–0.85**	**0.47**	**0.35–0.63**	1.31	0.97–1.77
Town^b^	0.93	0.72–1.19	**0.70**	**0.53–0.93**	1.16	0.89–1.51
Education high^c^	0.90	0.70–1.17	**0.63**	**0.48–0.84**	**1.80**	**1.34–2.41**
Education low^ c^	1.38	0.85–2.23	1.70	0.79–3.67	**0.60**	**0.37–0.97**
Professionals^d^	0.86	0.65–1.13	1.05	0.78–1.42	1.04	0.76–1.42
Workers^d^	1.07	0.84–1.36	1.31	1.00–1.72	0.98	0.76–1.27
Retired^d^	0.61	0.30–1.22	0.62	0.24–1.65	0.87	0.46–1.67
Income high^e^	0.81	0.57–1.14	0.99	0.68–1.43	0.80	0.54–1.17
Income low^ e^	0.93	0.76–1.14	1.16	0.93–1.44	1.06	0.85–1.32
Regular visits ^f^	**0.37**	**0.31–0.45**	**0.62**	**0.51–0.77**	**1.99**	**1.60–2.48**
Tooth brushing^†^	**0.75**	**0.64–0.88**	0.88	0.74–1.06	**1.29**	**1.09–1.52**
Molar region						
Age^†^	**0.968**	**0.96–0.976**	**1.099**	**1.08–1.118**	**0.959**	**0.949–0.968**
Female^a^	0.93	0.77–1.12	**1.92**	**1.37–2.69**	**1.33**	**1.05–1.68**
Capital^b^	1.22	0.91–1.63	0.79	0.47–1.31	**1.46**	**1.02–2.09**
Urban centre^b^	0.89	0.70–1.13	0.76	0.48–1.19	**1.38**	**1.02–1.87**
Town^b^	0.91	0.73–1.15	0.89	0.54–1.47	1.30	0.99–1.70
Education high^c^	0.89	0.71–1.13	0.74	0.51–1.07	**1.36**	**1.00–1.83**
Education low^ c^	1.18	0.73–1.90	3.05	0.4–23.47	**0.35**	**0.21–0.56**
Professionals^ d^	0.80	0.62–1.02	1.07	0.72–1.57	1.13	0.82–1.56
Worker^d^	0.83	0.66–1.04	1.13	0.74–1.72	0.95	0.74–1.24
Retired^d^	**0.23**	**0.11–0.49**	0.27	0.03–2.29	0.57	0.29–1.12
Income high^e^	0.92	0.67–1.25	0.86	0.54–1.37	0.79	0.52–1.19
Income low^ e^	1.17	0.97–1.40	0.88	0.65–1.20	**0.70**	**0.56–0.88**
Regular visit^f^	**0.46**	**0.38–0.55**	0.96	0.71–1.28	**1.94**	**1.55–2.44**
Tooth brushing^†^	**0.82**	**0.71–0.95**	**0.75**	**0.57–0.98**	1.16	0.98–1.38

* Dichotomized Decayed_ratio_ (“decayed”/“decayed”+“missing”+“filled”), Missing_ratio_ (“missing”/“decayed”+“missing”+“filled”), Filled_ratio_ (“filled”/“decayed”+“missing”+“filled”). Cut–off points: no “decayed” teeth present versus one or more “decayed” teeth present (Decayed_ratio_); no “missing” teeth versus one or more teeth “missing” (Missing_ratio_); no “filled” teeth present versus one or more “filled” teeth present (Filled_ratio_). ^†^Age and Tooth brushing—numerical variables. References (OR = 1) respectively: ^“a”^male; ^“b”^rural settlements (small towns and villages); ^“c”^education middle; ^“d”^occupational status intermediate; ^“e”^income middle; ^“f”^irregular visits. Bold figures indicate significant relationship.
